# Diagnosis of Severe Fetal Anemia Based on Perinatal Outcomes: A Comparative Analysis of the Current Reference Values

**DOI:** 10.1155/2013/351258

**Published:** 2013-11-20

**Authors:** Zilma Silveira Nogueira Reis, Gabriel Costa Osanan, Tiago Lanfernini Ricardo Coelho, Cezar Alencar De Lima Rezende, Henrique Vitor Leite, Antônio Carlos Vieira Cabral

**Affiliations:** ^1^Department of Gynecology and Obstetrics, Federal University of Minas Gerais, Brazil; ^2^Obstetrics and Gynaecology Department, Universidade Federal de Minas Gerais (UFMG), Avenida Professor Alfredo Balena, 190, Funcionários, Belo Horizonte, 30.130.100 Minas Gerais, Brazil; ^3^Federal University of Minas Gerais, Brazil

## Abstract

*Objectives*. To compare current criteria for severe fetal anemia diagnosis. *Methodology*. A cohort study analyzed 105 alloimmunized fetuses that underwent cordocentesis due to risk of anemia. Concordance among the diagnostic criteria for severe fetal anemia, hemoglobin deficit >7 g/dL, hemoglobin deficit ≥5 g/dL, and hemoglobin concentration <0.55 MoM, was analyzed using Cohen's Kappa index. Perinatal mortality, fetal hydrops, and fetal acidosis were used to discuss discordances. *Results*. There was fair concordance among the three criteria analyzed: 0.80 (Kappa index, IC 95%: 0.67 to 0.93) when comparing hemoglobin deficit >7.0 g/dL and hemoglobin concentration <0.55 MoM criteria, 0.63 (Kappa index, IC 95%: 0.47 to 0.69) when comparing hemoglobin deficit ≥5.0 g/dL and hemoglobin deficit >7.0 g/dL reference, and 0.77 (Kappa index, IC 95%: 0.64 to 0.90) when comparing hemoglobin deficit≥5.0 g/dL and hemoglobin concentration <0.55 MoM standards. Eighteen cases were classified differently depending on the criteria used. The cut-off point of hemoglobin deficit ≥5 g/dL was the best criterion to discriminate fetuses with poor perinatal outcome in our study. *Conclusions*. Relevant discordances in classification of severe fetal anemia were pointed out. Some criteria may underestimate the real gravity of fetal anemia.

## 1. Introduction

Maternal alloimmunization still affects a large number of pregnancies, particularly in developing countries [1, 2]. These pregnancies need specific follow-up at tertiary referral centers to carry out proper monitoring, in the view of a high risk of perinatal morbidity and mortality [3, 4]. When severe fetal anemia is suspected by a noninvasive method, cordocentesis is necessary to assess fetal hemoglobin concentration and then to determine the need of an intrauterine transfusion (IUT) [5, 6]. In this context, perinatal outcome also will depend on timely diagnosis and treatment of fetal anemia. For severely anemic fetuses the transfusion therapy is a life-saving procedure [7–9]. However, IUT carries risks for both mother and fetuses. In this way, it is important to determine which fetus is anemic and so it will need an IUT [7–9].

In this high-risk context, assessment of the degree of fetal anemia is an essential strategy for managing these pregnancies [10]. There are three main references for diagnosis and classification of fetal anemia. The first one was proposed by Nicolaides et al., published in 1998 [11]. These criteria use fetal hemoglobin deviation or deficit (mean hemoglobin for gestational age minus measured hemoglobin) as parameter for determining the severity of anemia. The authors consider as severely anemic fetuses those at high risk of hydrops that would generally occur when the hemoglobin deficit was >7 g/dL.

The second criterion was proposed by Bahado-Singh et al., published in 1998 [12]. These authors also use the concept of hemoglobin deficit as a parameter to classify the severity of anemia. They define severe anemia as a hemoglobin deficit ≥ 5 g/dL, since the risk of fetal hydrops is often below this below this value [13].

And finally, the third criterion was described by Mari et al., published in 2000. This author uses a different parameter for diagnosis and classification of fetal anemia, the multiples of median (MoM) of hemoglobin levels (calculated by dividing the measured hemoglobin value by the expected value for gestational age). Mari et al. define, as severely anemic fetuses, the presence of fetal hemoglobin concentration of less than 0.55 MoM at a blood cord sample [10]. This cut-off point was also based on the risk of fetal hydrops.

In this manner the purpose of this study was to make a comparative analysis of these three criteria used to diagnose and classify fetal anemia, based on perinatal outcome of fetuses followed and treated at a university referral center for Rh alloimmunization in Brazil.

## 2. Patients and Methods

A cohort study analyzed 151 fetuses that underwent cordocentesis due to suspected anemia. Fetal blood was sampled from January 1999 to December 2009, at the Fetal Medicine Center at the Clinical Hospital of the Federal University of Minas Gerais, a tertiary referral center for alloimmunized pregnancies in Brazil. Clinical records were retrospectively accessed. The study was approved by UFMG Research Ethics Committee. 

Eligibility criteria for inclusion in the study were fetuses submitted to cordocentesis due to the risk of anemia, access to information in medical records such as fetal hemoglobin levels before first cordocentesis; and reliable gestational age, confirmed by the latest reliable menstruation date and by an ultrasound examination performed at 20 weeks of gestation. 

**Figure 1:**
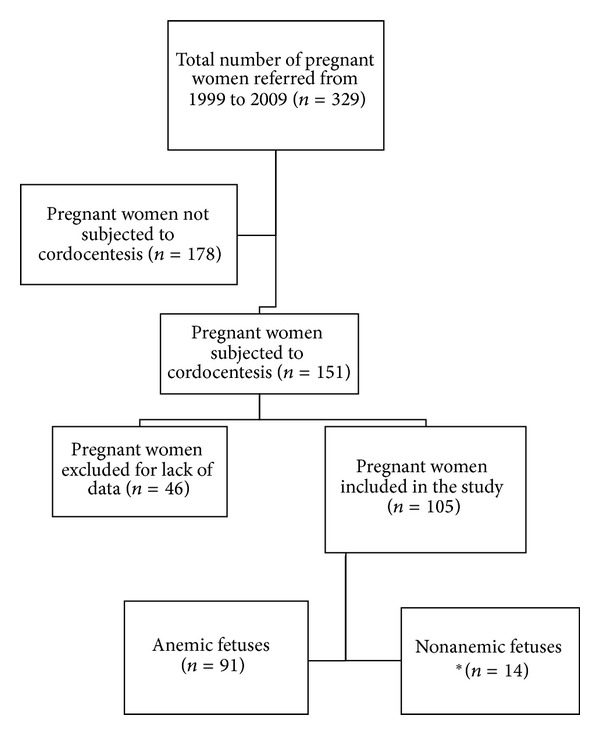
Flow diagram of enrolled fetuses at risk of fetal anemia from Rh.

During this 11-year period, 329 pregnancies were referred to our Fetal Medicine Center due to rhesus alloimmunization (Figure 1). From this total, 151 (45.9%) needed cordocentesis to confirm fetal anemia and to evaluate the need of IUT. From these 151 cases, 46 were excluded from this analysis due to unclear or insufficient clinical records (40 cases had unreliable gestational age at first cordocentesis and six medical records had no information about hemoglobin values at the first IUT). 

Cordocentesis was indicated, between 19 and 34 weeks of gestation, based on previous obstetric history, indirect Coombs test titer, presence of fetal hydrops, or altered cardiofemoral index (CFI) and peak systolic velocity middle cerebral artery (MCA-PSV) [13, 14]. Fetal hemoglobin concentration was measured using the HemoCue, a photometric technique on drops of blood taken immediately before the transfusion. In addition, the blood was sent for confirmation of the hemoglobin concentration measured using conventional techniques and for determination of fetal gasometry. The hemoglobin deficit was calculated based on the difference between the hemoglobin expected for the gestational age and that found in the puncture. IUT was performed when the fetal hemoglobin concentration deficit was ≥5 g/dL [12]. 

Finally we analyzed concordance among the criteria proposed by Nicolaides et al. [11], Bahado-Singh et al. [12], and Mari et al. [10] (Table 3), by means of diagnosis of severe anemia and prediction of perinatal outcome.

For the statistical descriptive analysis, variables were presented using central tendency and dispersion measures, according to the nature of their frequency distribution and absolute and relative frequencies.

In order to provide better understanding of the concordances and discordances among the diagnostic criteria of anemia, the fetal hemoglobin levels were plotted on a graph superimposed on the reference criteria curves. The Interrater agreement among the three criteria (for diagnosis of severe anemia) was obtained by Cohen's Kappa index, with respective confidence intervals of 95%. For this purpose references for fetal severe anemia diagnosis were compared. 

Perinatal outcome parameters such as pH value and hydrops [15] at first cordocentesis, perinatal mortality, and 5th minute APGAR index were compared to the criteria. After comparing the current standards, the cases were distributed into three categories of agreement: concordant severe anaemia cases, concordant nonsevere anaemia cases, and discordant severe anaemia cases. 

To detect differences in perinatal outcome among the groups the nonparametrical Kruskal-Wallis and Pearson's Chi-square test were utilized. Statistical significance was defined as *P* < 0.05. Statistical analysis calculations were performed using MINITAB Release 14.12.0 1972–2004 Minitab Inc. software.

## 3. Results

Most of the pregnant women (64.8%, *n* = 68) arrived at this university center during third trimester of gestation. The hydrops incidence at first cordocentesis was 24.8% (*n* = 26). In these cases, 80.6% (*n* = 122) of pregnant women showed an indirect Coombs test titre ≥ 1 : 64. The characterization of the pregnancies evaluated in the study was presented in Table 1. The lack of a specific Rh immunoprophylaxis, at birth and at miscarriage, was the major cause of maternal sensitization (89.9%, *n* = 95). The most prevalent erythrocyte antibody found in this study was the anti-D (87%, *n* = 86/99) (Table 1). The perinatal mortality rate found was 19.1% (*n* = 20). Severe fetal anemia frequency ranged from 22.9% to 39.1%, depending on the diagnosis criterion adopted (Table 1).

**Figure 2:**
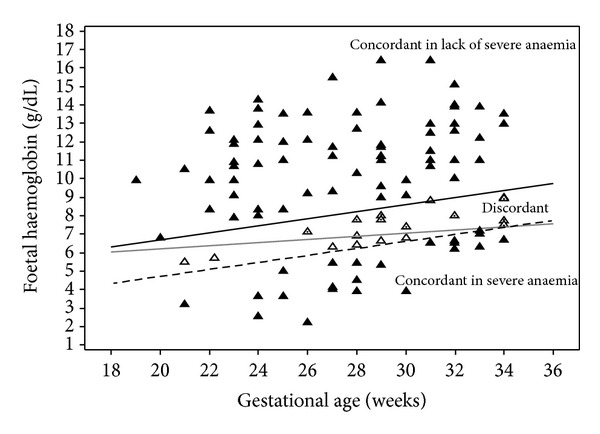
Fetal hemoglobin concentration values assessed at first cordocentesis, plotted under the severe anemia diagnosis threshold, according to the anemia different criteria. Solid triangles: concordant cases. Hollow triangles: discordant cases.

The fetal hemoglobin concentration values, according to the respective gestational age, were plotted on a graph with the reference standards (the three criteria) for fetal anemia (Figure 2). Eighteen fetuses could be classified as nonseverely or severely anemic, depending on the criterion employed (hollow triangles).

Concordance on the diagnosis of severe anaemia was 0.80 (Kappa index, IC 95%: 0.67–0.93) when comparing the criteria of Nicolaides et al. (hemoglobin deficits >7.0 g/dL) and Mari et al. (hemoglobin concentration <0.55 MoM) [11, 12], even though seven diagnoses were discordant.

Moderate concordance (0.63, Kappa index, IC 95%: 0.47–0.69) was observed when comparing Bahado-Singh et al. (hemoglobin deficit ≥5.0 g/dL) [12] and Nicolaides et al. (hemoglobin deficit >7.0 g/dL) [11] criteria for severe anemia. However, 18 diagnoses were discordant.

Finally, fair concordance was observed: 0.77 (Kappa index, IC 95%: 0.64–0.90) comparing Bahado-Singh et al. (hemoglobin deficit ≥5.0 g/dL) and Mari et al. (hemoglobin concentration <0.55 MoM) criteria. All the 30 cases considered severely anemic by Bahado-Singh et al. (hemoglobin deficit ≥5.0 g/dL) [12] criteria were also classified as severely anemic by Mari et al. (hemoglobin concentration <0.55 MoM) [10]. Once more, 11 cases showed discordant classification. 

Perinatal results were presented in Table 2. The outcome was characterized by high mortality (20%) and high incidence of hydrops (33.3%). Weight at birth ranged from 390 to 3180 g (median 2180 g). Only the hemoglobin deficit ≥5.0 g/dL criterion for severe anemia [12] could identify all cases with poor perinatal outcome.

As mentioned before, when comparing the current criteria for the diagnosis of severe anemia (two at each time), the cases could be distributed into three categories: the concordant severe anemia cases, the concordant nonsevere anemia cases, and the discordant severe anemia cases (Table 2). The APGAR score and the pH values at cord blood sample (at first cordocentesis) were similar among these groups (Table 2). However, the presence of hydrops and perinatal death were statistically higher in the group of concordant severe anemia cases than in the group of concordant nonsevere anemia (Table 2). 

In this study, the standard that uses a cut-off point of hemoglobin deficit ≥ 5 g/dL [12] to define severe fetal anemia was the only criterion available to classify as severely anemic fetuses, all the cases that had poor perinatal outcome.

## 4. Discussion 

The absence of previous studies that compare current criteria for the diagnoses of severe fetal anemia coexists with an uncritical clinical use of them. This study provides a retrospective analysis that compares these three standards to the perinatal outcome of pregnancies complicated by maternal alloimmunization at our service. We also highlight the potential discrepancies among these three criteria when classifying fetuses as severely anemic. It is important to emphasize that this study was not addressed to evaluate recommendations to indicate cordocentesis or to compare the noninvasive methods to predict fetal anemia or even to analyze current protocols to manage Rh-alloimmunized pregnancies [2, 14]. 

By pointing out concordances and discordances in classifying the severe anaemia, this study may help to define protocol standards for the management of pregnancies complicated by maternal alloimmunization. It is important to note that such a comparative approach was possible, just because it took place at a referral center for the care of alloimmunized pregnancies. However, as all retrospective studies, some peculiar limitations were present in this analysis. An important one referred to the changes (worldwide) in the protocol for handling these pregnancies, especially when the MCA-PSV [10] substituted the amniocentesis in the prediction of fetal anemia. This fact reduced drastically the utilization of invasive procedures for diagnosing fetal anemia. The MCA-PSV measurement by ultrasound turned safer prediction of fetal anemia in these pregnancies and then reduced the risk (including fetal death) related to unnecessary cordocentesis. Furthermore, the experience of our service with another noninvasive method, the CFI [8, 9, 16], added a new and important parameter to be used in association with the MCA-PSV to predict important fetal anemia. Finally the present study faced another limitation related to the incompleteness of data (due to information not inserted into the records) or even the loss of cases, leading to exclusions.

The first study to propose a classification for fetal anemia in a group of alloimmunized pregnancies was performed by Nicolaides et al. [11]. To achieve it, the authors determined a normal reference range of fetal hemoglobin, based on 210 samples of umbilical cord blood from conceptuses without anemia, undergoing prenatal diagnosis. Subsequently, they compared the normal range of hemoglobin concentration to the levels found in umbilical cord from alloimmunized fetuses, in a way that they could evaluate severity of anemia. Thus the authors defined severe anemia as the presence of hemoglobin deficit >7 g/dL, since hydropic fetuses in their study had a hemoglobin concentration 7 to 10 g/dL below the normal mean for gestational age. This criterion is probably the most traditional reference to define and classify fetal anemia nowadays. However, in our study, some fetuses with markers of poor perinatal outcome were not classified as severely anemic by this reference. 

The second criterion studied was the one created by Bahado-Singh et al. [12]. These authors, based on the references established by Nicolaides et al. [11], decided to reduce the cut-off for the diagnosis of severe anemia to a hemoglobin deficit ≥5 g/dL, in the attempt to increase sensitivity to detect severely anemic cases, and so to ensure that the anemic fetuses would be treated before development of hydrops. Moreover, he classifies severity of anemia in only two groups: mild and severe anemic fetuses. In the present study, all conceptuses that showed hydrops or died during the perinatal period were classified as severely anemic by this criterion.

The third and last criterion evaluated was defined by Mari et al. [10]. This author, as Nicolaides et al. [11], determined the normal range of hemoglobin concentration from his own population of fetuses [10, 14] and classified fetal anemia into mild, moderate, and severe anemia. Differently from Nicolaides et al. [11], Mari et al. [10] found that hemoglobin concentration increases exponentially with advancing gestation and so they decided to classify severity of anemia by means of MoM (multiples of median) for haemoglobin concentration (in order to adjust for the effect of gestational age on the measurement). With this approach, the authors demonstrated that fetuses with severe anemia had a hemoglobin value <0.55 MoM for a given gestational age [10, 14]. In our analyses, unfortunately this definition of severe fetal anemia also did not include all fetuses with significant markers of poor perinatal outcome. 

Evaluating the different criteria of severe anemia diagnosis, we found that there are important divergences capable of modifying perinatal outcome, especially regarding the occurrence of fetal hydrops and perinatal mortality. On comparing Bahado-Singh et al. [12] and Mari et al. [10] criteria, it was possible to observe 11 divergent cases. Out of these, three cases (30%) were classified as having a severe form of anemia by Bahado-Singh et al. [12] but not by Mari et al. [10], progressed to perinatal death. The same discordance happened with the other two cases (20%) that were hydropic at the first cordocentesis and were not considered severely anemic by Mari standards [10]. Also on comparing Bahado-Singh et al. [12] and Nicolaides et al. [11] standards, 18 fetuses were divergent in severe anemia classification were observed. At this time, five cases (27.8%) were classified as having a severe form of anemia by Bahado-Singh et al. [12] but not by Nicolaides et al. [11], progressed to perinatal death. Moreover other six (33%) that were hydropic would not considered severely anemic by Nicolaides et al. [11] cut-off point. 

Among the three recommendations, we believe that defining severe anemia in the presence of hemoglobin deficit ≥5 g/dL [12] could offer more safety intervention by allowing earlier and more timely treatment for these conceptuses, despite its risk. It is necessary for other prospective clinical trials to confirm our findings. In any event, we hope that this study may contribute to a better management of pregnancies complicated by maternal alloimmunization.

## Figures and Tables

**Table 1:** Characteristics of alloimmunized pregnancies included in the study.

Gestational and perinatal characteristics	*n*	Values	Variation or %
Maternal age, years (mean ± SD)	105	29.7 ± 5.3	19 to 43
Parity (median, range)	105	4	1 to 11
Anti-D antibodies (alone), *n*(%)	99	54	54.5%
Anti-D + anti-C antibodies, *n*(%)	99	32	32.5%
GA, weeks, at first cordocentesis (mean ± SD)	105	27.9 ± 3.9	19 to 34
Fetal hemoglobin (g/dL), at first cordocentesis (mean ± SD)	105	9.4 ± 3.4	2.2 to 16.4
Fetal severe anemia (>7.0 g/dL hemoglobin deficit)*, *n*(%)	105	24	22.9%
Fetal severe anemia (≥5.0 g/dL hemoglobin deficit)**, *n*(%)	105	41	39.1%
Fetal severe anemia (hemoglobin < 0.55 MoM***, *n*(%)	105	30	28.6%
Fetal pH, at first cordocentesis (median, range)	101	7.35	7.07–7.49
Fetal hydrops at first cordocentesis, *n*(%)	105	26	24.8%
Perinatal mortality, *n*(%)	105	20	19.1%
Apgar 5 (median, range)	89	9	2 to 10

*Nicolaides et al. [11], **Bahado-Singh et al. [12], and ***Mari et al. [10]; SD: standard deviation, GA: gestational age.

**Table 2:** Perinatal outcome, according to concordance in classifying anemia severity using the several criteria analyzed.

	*N*	Concordant nonsevere anemia cases	Discordant severe anemia cases	Concordant severe anemia cases	*P*
hb ≥ 5.0 g/dL deficit [12] versus hb < 0.55 MoM [10]		(n = 64)	(n = 11)	(n = 30)	

Fetal pH^#^	101	7.39 (7.10–7.49)	7.33 (7.19–7.43)	7.35 (7.07–7.45)	0.076*
Fetal hydrops^+^	105	8 (12.5%)	2 (20%)	16 (51.6%)	<0.001**
Perinatal death	105	5 (7.8%)	3 (30%)	12 (38.7%)	0.001**
Apgar 5	89	9 (2–10)	9 (9-9)	9 (2–10)	0.603*

hb > 7.0 g/dL deficit [11] versus hb ≥ 5.0 g/dL deficit [12]		(*n* = 64)	(*n* = 18)	(*n* = 23)	

Fetal pH^#^	101	7.39 (7.10–7.49)	7.34 (7.19–7.46)	7.34 (7.07–7.45)	0.069*
Fetal hydrops^+^	105	8 (12.5%)	6 (33.3%)	12 (52.2%)	0.001**
Perinatal death	105	5 (7.8%)	5 (27.8%)	10 (43.5%)	0.001**
Apgar 5	89	9 (2–10)	9 (9-9)	9 (2–10)	0.318*

hb > 7.0 g/dL deficit [11] versus hb < 0.55 MoM [10]		(*n* = 74)	(*n* = 8)	(*n* = 23)	

Fetal pH^#^	101	7.38 (7.10–7.49)	7.37 (7.30–7.43)	7.34 (7.07–7.45)	0.172*
Fetal hydrops^+^	105	10 (13.5%)	4 (50%)	12 (52.2%)	<0.001**
Perinatal death	105	8 (10.8%)	2 (25%)	10 (43.5%)	0.002**
Apgar 5	89	9 (2–10)	9 (9-9)	9 (2–10)	0.539*

Note: ^#^1st cordocentesis, *Kruskal-Wallis test, **Chi-square test, hb: foetal hemoglobin value at first cordocentesis.

**Table 3:** Criteria for diagnosis and classification of fetal anemia.

	Mild anemia	Moderate anemia	Severe anemia
Nicolaides et al. [11]	Hemoglobin deficit <2 g/dL	Hemoglobin deficit ≥2 g/dL to 7 g/dL	Hemoglobin deficit >7 g/dL
Bahado-Singh et al. [12]	Hemoglobin deficit ≥2 g/dL to less than 5 g/dL	—	Hemoglobin deficit ≥5 g/dL
Mari et al. [10]	Hemoglobin concentration from 0.84 to 0.65 MoM	Hemoglobin concentration from less than 0.65 to 0.55 MoM	Hemoglobin concentration less than 0.55 MoM
